# Artemisinin Improved Neuronal Functions in Alzheimer's Disease Animal Model 3xtg Mice and Neuronal Cells via Stimulating the ERK/CREB Signaling Pathway

**DOI:** 10.14336/AD.2019.0813

**Published:** 2020-07-23

**Authors:** Xia Zhao, Shuai Li, Uma Gaur, Wenhua Zheng

**Affiliations:** Center of Reproduction, Development & Aging and Institute of Translation Medicine, Faculty of Health Sciences, University of Macau, Taipa, Macau, China

**Keywords:** Alzheimer's disease, Artemisinin, cognitive behavior, amyloid beta, 3×Tg mice, SH-SY5Ycells

## Abstract

The most common form of dementia is Alzheimer’s disease which is characterized by memory loss and cognitive disorders. The pathogenesis of Alzheimer’s disease is not known at present but toxicity of amyloid-beta is one of the central hypotheses. Amyloid-beta can stimulate the production of reactive oxygen species (ROS), cause oxidative stress, damage mitochondrial, cause inflammatory reactions and activate apoptosis related factors which lead to the neuronal death. In this study, we found that artemisinin, a first line antimalarial drug used in clinic for decades, improved the cognitive functions in Alzheimer’s disease animal model 3xTg mice. Further study showed that artemisinin reduced the deposition of amyloid-beta and tau protein, reduced the release of inflammation factors and apoptosis factors, and thereby reduced the neuronal cell death. Western blot assay showed that artemisinin stimulated the activation of ERK/CREB signaling pathway. Consistent with these results, artemisinin concentration-dependently promoted the survival of SH-SY5Y cell against toxicity of amyloid-beta protein 1-42 induced ROS accumulation, caspase activation and apoptosis. Artemisinin also stimulated the phosphorylation of ERK1/2 and CREB in SH-SY5Y cells in time and concentration-dependent manner. Inhibition of ERK/CREB pathway attenuated the protective effect of artemisinin. These data put together suggested that artemisinin has the potential to protect neuronal cells *in vitro* as well as *in vivo* animal model 3xTg mice via, at least in part, the activation of the ERK/CREB pathway. Our findings also strongly support the potential of artemisinin as a new multi-target drug that can be used for preventing and treating the Alzheimer’s disease.

Alzheimer's disease (AD) is the most widely occurring neurodegenerative disorder which is characterized by loss of memory and behavior disorders [1, 2] . According to the recent statistics, there are more than 44 million dementia patients worldwide in 2017 and the number will rise to 131.5 million by 2050, which will cause a serious social and economic burden. Deposition of amyloid plaques, neurofibrillary tangles (NFTs) and the neuronal loss are the three hall markers of AD [3-7]. In addition, oxidative stress, inflammation, mitochondria dysfunction and increased cholesterol levels are all mechanisms that have been associated with Alzheimer's disease [8, 9]. Among them deposition of β-amyloid is believed to be the principal component of senile plaques which are responsible for neuronal death and symptom of AD. β-amyloid is produced by hydrolysis of amyloid precursor protein (APP) by β-secretase and γ-secretase. Studies have found that β-amyloid can stimulate the production of reactive oxygen species (ROS), cause oxidative stress, damage mitochondrial, and activate apoptosis-related proteins and factors which lead to the neuronal death [10, 11]. In addition, Aβ can also cause neuronal apoptosis by causing inflammatory reactions and neurofibrillary tangles in the brain, which is an important cause of AD formation and development [12, 13]. So far, neuroprotective strategies based on the amyloid hypothesis are still the central topic of research. In the amyloid hypothesis, the continuous production and reduced clearance of Aβ in neurons lead to the accumulation of these toxic peptides which ultimately leads to a series of pathological events in the process of AD. Many reports suggest that aggregation of Aβ oligomers and fibrils induce the neurotoxicity and lead to the neurodegeneration [14, 15]. Neuronal death and synaptic damage during AD induced by Aβ caused the dysfunction of different types of neurons such as cholinergic neurons and also lead to the cognitive deficits [16].

Current treatments are majorly symptomatic utilizing cholinesterase inhibitors such as rivastigmine, galantamine, and donepezil [17]. At moderate as well as severe stages another drug which ismemantine, an N-Methyl-Daspartate receptor antagonist, is also used for AD treatment [18, 19]. However, the effects of current treatments are far from satisfactory and there is no effective treatment for AD at present. Defects in the past approaches of treating AD are: unacceptable toxicity and side effect; single drugs act on single mechanism; treating late stage disease. Therefore, finding multi-target drugs may be an effective strategy for the treatment of AD.

Artemisinin (ART) is extracted from the plant Artemisia annua. It is a very effective anti-malarial drug which is used for the treatment of malaria in clinics for decades and saved millions of malaria patients worldwide [20, 21]. ART can pass the blood-brain barrier (BBB) with no observable side effects. In addition, artemisinin has shown to have antioxidant, anti-inflammatory, anti-viral and anti-bacterial effects which exhibit a variety of beneficial pharmacological effects outside of the nervous systems [23]. More important is that we have recently shown that artemisinin has significant neuroprotective effects, which have not been explored yet [22-23, 35]. There is little research on the protective effect of artemisinin on the diseases of the central nervous system (CNS), especially on AD, although artemisinin was suggested to extenuate amyloidogenesis by anti-inflammation [24]. Moreover, the therapeutic effects of artemisinin on AD and the underlying mechanisms are still very elusive.

Transgenic animal models of AD are important tools to further study and evaluate the effect of new chemical/drug on AD. Many animal models of AD have been developed, among which 3xTg mice is the best model that closely mimic the feature of AD. 3xTg mouse is a triple transgenic mouse having mutant forms of APP_Swe_, Tau_P301 L_ and PS1_M146V_. The time-course pattern of the region in which neuropathology occurs in 3xTg mice closely mimics AD-related changes [25-27]. Long-term memory dysfunction began to appear at 4 months of age. At 6 months of age, the level of oxidative stress is significantly increased, extracellular amyloid deposition occurred, and short-term memory function was impaired. Key enzymes of mitochondrial oxidative phosphorylation showed significant decrease in activity at 9 months of age. At 12 months of age, mitochondrial respiratory efficiency decreased significantly, and obvious amyloid deposition and conformational changes of Tau protein appeared in the cortex and hippocampus [28-31]. Compared to other mice models, 3xTg mouse is the best models that closely mimic the features of AD. It helps to clarify the relationship between amyloid, neurofibrillary tangles, neuroinflammation in the same model and is the best model for studying multi-target drugs. Therefore, 3xTg mice could be an ideal model to study AD and its underlying mechanisms.

In this study, we found that ART significantly improved the cognitive ability, improved neuronal functions by reducing oxidative stress and release of inflammatorily factors and apoptosis-related proteins and factors in 3xTg mice. In addition, ART reduced the deposition of Aβ plaques and neurofibrillary tangles in 3xTg mice. Finally, ART reversed the phosphorylation of ERK/CREB in 3xTg AD mice brain. Experiments using SH-SY5Y also indicated that ART promoted the survival of neuronal cells against Aβ induced impairment via the ERK/CREB pathway. All these findings support that ART can be act via multiple targets and can be used as a potential drug for the prevention and treatment of AD.

## MATERIALS AND METHODS

### Reagents and chemicals

Analytical grade artemisinin was purchased from Meilunbio (Dalian China). Dimethyl sulfoxide (DMSO) and sucrose were procured from Sigma-Aldrich (St. Louis, MO, USA). Hoechst 33258, 5,5′,6,6′-tetrachloro-1,1′,3,3′-tetraethyl-benzimidazolyl-carbocyanineiodide (JC-1), and Poly-D-lysine, 3-(4,5-Dimethylthiazol-2-yl)-2,5-diphenyltetrazolium bromide (MTT), were obtained from Molecular Probes (Eugene, OR, USA). CellROX Deep Red Reagent was ordered from Thermo Fisher Scientific (Rockford, IL, USA). DCFH-DA reagent was ordered from Beyotime Institute of Biotechnology (Shanghai, China). Annexin V-FITC/PI Apoptosis Detection Kit was obtained from BD Biosciences (San Diego, CA, USA). Trypsin (0.25%) and fetal bovine serum (FBS) and 0.25% were procured from Life Technologies (Grand Island, NY, USA). MEK/ERK inhibitor PD98059 was ordered from Calbiochem (San Diego, CA, USA). The information of antibodies was shown in Table 1. The detail information of β-Amyloid (1-42) was provided in Table 2

**Table 1:** Antibody Information.

Antibody	Cat. NO	Source	Company
APP/β-Amyloid (NAB228)	2450	Mouse	CST
β-Amyloid (1-42 Specific) (D3E10)	12843	Rabbit	CST
Tau (Tau46)	4019	Mouse	CST
Phospho-Tau (Ser396)	11102	Rabbit	SAB
phospho- Tau (thr212)	1343547	Rabbit	CALHIOCHEM
Phospho- Erk1/2 (Thr202/Tyr204)	9101S	Rabbit	CST
ERK 1/2 Polyclonal	40902	Rabbit	SAB
BAX	34260-2	Rabbit	SAB
Bcl-2	32012	Rabbit	SAB
Cleaved-Caspase3	9661	Rabbit	CST
Cleaved-Caspase9	7327	Rabbit	CST
Caspase-1	2225s	Rabbit	CST
Caspase-3	9662	Rabbit	CST
Caspase9	9508	mouse	CST
Cytochrome c (136F3)	4280	Rabbit	CST
GFAP (GA5)	3670	Mouse	CST
IL-1beta(3A6)	12242s	Mouse	CST
Pro-IL-1β Polyclonal Antibody	41059	Rabbit	SAB
Phospho-CREB (Ser133) (87G3)	9191	Rabbit	CST
Phospho-c-Jun (Ser73)	9164	Rabbit	CST
AIF(Iba1)	38603	Rabbit	SAB

SAB (Signalway antibody).

### Animal and treatment

The homozygous 3xTg AD mice (APP_Swe_, Tau_P301 L_ and PS1_M146V_ transgenes) was bought from the Jackson Laboratory and retained in the Animal Facility of University of Macau. All animal experiments were performed in accordance with guidelines accepted by the University of Macau Animal Ethics Committee (protocol No.:UMAEC-13-2015) [16]. The animal housing conditions were regulated (temperature 23 °C; humidity 60-65%; light from 7:00 to 19:00), and water and food were readily accessible.

3xTg mice (12 month old, male n=6, weight 32-35g) were randomly segregated in five groups: Control (WT mice+ PBS), 3xTg (3xTg mice+ PBS), 3xTg + 1mg/kg ART, 3xTg + 5mg/kg ART, 3xTg + 10mg/kg ART. ART was dissolved in 2% DMSO in PBS. For one month all the groups were treated with corresponding solutions everyday by intraperitoneal injection.

### Morris water maze (MWM)

The Morris water maze (MWM) test is the classic behavioral experiment which tests the learning and memory ability [32]. The MWM was procured from ZS Dichuang (Beijing, China) having a xeye Aba video tracking system. The MWM is a circular pool (120 cm in diameter and 60 cm in height with white bottom and wall), 1cm under the water surface, a white circular platform (diameter 8 cm; height 30 cm) was submerged. Food-grade titanium dioxide (100 g) was added into the tank to whiten the water so that the mice cannot visually recognize the platform. And the temperature was maintained at 25 °C. On each side of the wall of the quadrants distinct colored papers were placed as visual positional hint. The video tracking camera was staged onto the ceiling directly above the center of the pool to monitor subject swimming parameters [32].

For every training session, the mice were released to the maze back to back from four random points of the tank and were permitted to look for the platform for 60 s. In case when the mice did not find the platform within 60 s, they were securely directed to it. The time and movement route of the mice to find the platform were recorded. Mice were kept in a drying cage with a heating system during the test interval. The above operation was repeated for the 2nd, 3rd, 4th, and 5th day. The average latency in the 5-day navigational experiments of each group of mice was measured as an indicator for judging the learning ability of mice. On the 6th day, the platform was removed from the water maze, and the mice were allowed to swim freely for 60 s for space exploration experiments. The number of crossings of the platform, and the residence duration of each mouse in each quadrant of the platform were recorded.

**Table 2:** Detail information of β-Amyloid (1-42).

**Sequence** **(Three-Letter Code)**	H - Asp - Ala - Glu - Phe - Arg - His - Asp - Ser - Gly - Tyr - Glu - Val - His - His - Gln - Lys - Leu - Val - Phe - Phe - Ala - Glu - Asp - Val - Gly - Ser - Asn - Lys - Gly - Ala - Ile - Ile - Gly - Leu - Met - Val - Gly - Gly - Val - Val - Ile - Ala - OH
**One Letter Code**	DAEFRHDSGYEVHHQKLVFFAEDVGSNKGAIIGLMVGGVVIA

Molecular Formula: C_203_H_311_N_55_O_60_S Relative Molecular Mass: 4514.10

### Tissue preparation

After MWM test, the mice were put under anesthesia with pentobarbital sodium (50 mg/kg) and the blood was sampled from the eyeballs. Then, 1xPBS (pH7.4) was transcardially flushed in the mouse. Brains were quickly collected and dissected on ice, and the left hemisphere brain tissue were immersion fixed with 4% paraformaldehyde overnight, and embedded in paraffin following the standard procedures, which can be stored for a long time [33]. The cortexes of right hemisection were cut open on ice and kept at -80 °C for Western blot assay.

### Immunohistochemistry and Immunofluorescence

Immunohistochemistry (IHC) is used to localize/visualize the expression of protein in a mounted tissue section using protein specific antibodies [33]. The brain tissues were sliced into 5μm slices using Manual Rotary Microtome Basic Instrument (Leica RM2235, Leica, Germany). After routine dewaxing hydration, antigen retrieval was carried out by immersing in 0.01 M citrate buffer solution. This was followed by incubation with 3% H_2_O_2_ for 15 min to remove endogenous peroxidase activity. After blocking with 10% bovine serum albumin (BSA) for 1 h, the primary antibody was added dropwise to slices which were stored overnight at 4 °C. Next day, the brain tissue sections were then incubated with second antibody for 60 min and followed by DAB color development.

Immunofluorescence is a method in which a known antibody labeled with fluoresce in is used as a probe to detect a target antigen in a tissue or a cell to be tested, and the formed antigen-antibody complex is provided with fluorescein, and thus can be directly observed by a fluorescence microscope. The brain tissue was embedded in OCT (optimal cutting temperature) compound. The brain was sliced into 20 μm slices using a low temperature thermostat (Leica CM3050, Leica, Germany). Each section was washed with 1xPBS for three times and then blocked with 10%BSA for 1h at room temperature. After that, tissue sections were incubated with primary antibody in PBS containing 1% BSA at 4°C overnight. The following day, sections were incubated with appropriate secondary antibody for 1h at room temperature in the dark. Nuclei were counterstained with DAPI, and the images were acquired with a Nikon A1 confocal microscope.

### Congo red staining and Nissl staining

The embedded brain tissues were cut into 5μm thin slices and followed by dewaxing in xylene and dehydrating by alcohol gradients. After that, sections from each group were stained with Highman Congo red staining solution for 5 to 10 minutes followed by washing three times with water, and then transferred in hematoxylin staining solution for 1~2min, stained for the nucleus. Washed with distilled water, and differentiated and returned to blue. After staining, slices were dehydrated by fractional alcohols, transparent with xylene and mounted with Neutral gum.

Nissl staining was used to detect the surviving neurons. Nissl is large and large in number, indicating that nerve cells synthesize proteins with strong functions; on the contrary, when nerve cells are damaged, the number of Nissl bodies will decrease or even disappear. For each group, sections (each 5μm thick) were stained with Nissl staining solution (C0117, Beyotime, Shanghai, China) for 10 min at 37-50 °C and then washed, color separated, dehydrated, hyalinized and mounted. The cells which contained a clear body and nucleus were counted as surviving neurons in the sections of hippocampus. The survival index was measured following this formula: survival index (%) = (number of surviving neurons/total number of neurons) x 100%[34].

### TUNEL assay

Cell apoptosis was determined by the TUNEL assay performed using a TUNEL kit (C1098, Beyotime, Shanghai, China) following manufacturer’s instructions. Processed samples were incubated in a TUNEL reaction mixture (50 μL) for 60 min at 37 °C under dark conditions. Color developed in DAB coloring solution. Brown is apoptotic cells positive for TUNEL staining. Apoptosis index in the cortex area was measured using this formula: apoptosis index (%) = (apoptotic neurons/ total neurons) X 100%.

### MTT assay

MTT assay was used to determine the cell viability [35]. After treatment, the cells were further incubated with MTT (0.5 mg/ml) for 3-4h. The medium was removed from each well and 100μl DMSO was added to dissolve the dark-blue formazan crystals that were formed in living cells. The absorbance was measured in each well solution using a microplate reader (SpectraMax 250, Molecular Device, Sunnyvale, CA, USA).

### Annexin V-FITC/PI staining for apoptosis evaluation

Flow cytometry assay was performed following the kit guidelines. Briefly, SH-SY5Y cells were trypsinized after appropriate treatment, rinsed twice with ice-cold PBS, centrifuged at 1000 rpm for 5 min and re-suspended the cells in Annexin V-FITC/PI binding buffer (195 μL). Annexin V-FITC (5 μL) was supplemented and the cells were stored in dark at room temperature for 30 min. Cells were then pellet down by centrufing at 1000rpm for 5 min and re-suspended in Annexin V-FITC/PI binding buffer (190 μL); Propidium Iodide (PI) (10 μL) was further added, followed by incubation in the dark for 5mins. The apoptotic cells were quantified using Flow cytometer.

### Measurement of intracellular ROS levels

After appropriate treatment, the SH-SY5Y cells were stored at dark with CellROXs Deep Red Reagent (5 mM) in DMEM (without PBS) for 1h, rinsed twice with 1x PBS solution and the fluorescence was measured and recorded under an Infinite M200 PRO Multimode Microplate at an emission wavelength of 665 nm and an excitation wavelength of 640 nm.

### Measurement of mitochondrial membrane potential (△ψm)

JC-1 dye was utilized to observe the mitochondrial integrity. In brief, SH-SY5Y cells (6×10^3^ cells/well) were seeded in a 96-well plate with 1%FBS DMEM medium. After suitable treatment, the cells were incubated with 1x JC-1 (10 μg/ml in blank medium) at 37 °C for 30 min and rinsed two times with PBS. For signal quantification, the intensity of red fluorescence (excitation 560 nm, emission 595 nm) and green fluorescence (excitation 485nm, emission 535 nm) were measured and recorded using an Infinite M200 PRO Multimode Microplate. △ψm was calculated as the ratio of JC-1 red/green fluorescence intensity and the values were normalized to the control group.

### Mitochondrial / Cytosol protein extraction

Mitochondrial and Cytosol protein were isolated from SH-SY5Y cells and brain tissue using differential centrifugation as described by Ivan Dimauro et al [36]. After we got the supernatant (Cytosol) and pellet (crude mitochondria), the mitochondria was purified by using ultracentrifugation. The crude mitochondrial pellet was resuspended in 50 mM Tris-HCl (pH 7.4), 250 mM sucrose and protease inhibitors and ultracentrifuge at 100,000 g for 20 min at 4°C. The protein concentration was determined with BCA protein assay kit and analyzed by western blot using anti-cytochrome C antibody.

### Western blot

The shredded mice brain tissue was blended in RIPA buffer containing protease inhibitors. The tissue sample was disrupted by sonication, and then centrifuged at 12,000 g for 20 min at 4 °C, and the supernatants were taken out in separate tubes. Protein concentration of each sample was determined with a BCA Protein Assay [37]. Same amount of proteins was electrophoretically separated using polyacrylamide gel electrophoresis (PAGE) and transferred onto polyvinylidene fluoride (PVDF) membranes at 200 mA for 2h. The PVDF membranes containing protein bands were blocked with 5% skimmed milk at room temperature for 1h, and incubated with primary antibodies overnight at 4 °C. Following day, the membranes were incubated with horseradish peroxidase (HRP)-conjugated secondary antibodies (1:2000; CST) for 1h at room temperature. The specific protein bands were seen using the Bio-Rad Gel Doc XR documentation system.

### Statistical analysis

All the data was presented as mean ± SE. Each experiment was carried out in triplicates. For the MWM test, escape latency times in the hidden platform trial were analyzed via two-way ANOVA of repeated measures. Statistical differences were analyzed by one-way ANOVA in combination with post hoc Tukey’s test (α = 0.05) to assess the difference between any two groups by using GraphPad Prism 5.0 statistical software (GraphPad software, Inc., San Diego, CA, USA). P<0.05, P<0.01 were considered statistically significant.

**Figure 1.**
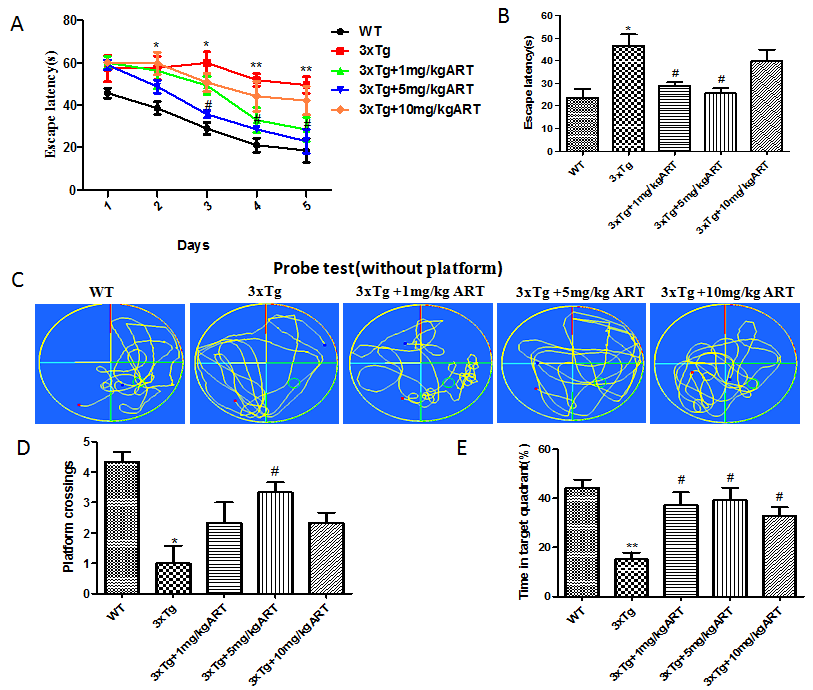
**Artemisinin improved the cognitive deficit of aged 3xTg mice**. **(A)** Average escape latencies curve (four trials per day in the five consecutive days). **(B)** Time needed to find the hidden platform (escape latency) during the training. **(C)** Representative path tracking in the probe tests without hidden platform in day 6. **(D)** The average crossing platform times of each group mice within 60s in day 6. **(E)** Time spent in the target quadrant where the platform had been located in day 6. * p<0.05 or **p<0.01 3xTg vs WT; #p <0.05 3xTg vs 3xTg+ART.

## RESULTS

### Artemisinin improved the cognitive function in 3xTg mice

In order to study the effect of artemisinin on cognitive functions of 3xTg AD mice, we performed MWM assay. Accordingly, one year aged 3xTg AD mice were divided to five groups and tested as indicated. Our results showed that artemisinin administration for 1 month in 3xTg AD mice significantly improved their ability to locate the platform on the MWM, and also resulted in enhancement in the spatial learning tasks. The average escape latencies for 5 consecutive days in each group are displayed in curves (Fig. 1A). The average escape latency of 3xTg + 5mg/kg ART group was significantly lower than 3xTg group in the 3rd, 4th and 5th day. Next, for quantificational evaluation (Fig. 1B), the 3xTg + 1mg/kg ART and the 3xTg + 5mg/kg ART group mice exhibited a higher retention performance on the learning test in comparison to 3xTg mice. The above results were further reinforced by a subsequent probe trial without the platform in the 6th day. The usual path tracking of each group in 60 s is displayed in Figure 1C. We computed the platform location crossing times and the percentage of target quadrant search time (Fig. 1D and Fig. 1E), both of which demonstrated the memory retention of the location of the hidden platform. The data indicated that there were more platform location crossing times and an increased percentage of target quadrant search time in the 3xTg+ART group in comparison of the 3xTg group. These results suggest that artemisinin can enhance the spatial memory capacity in AD mice.

**Figure 2.**
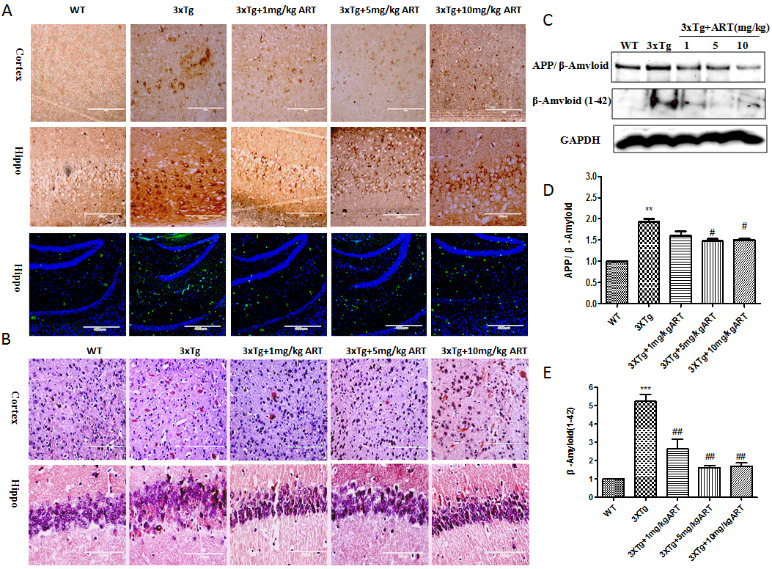
**Artemisinin reduced Aβ deposition in aged 3xTg mice**. **(A)** Immunohistochemistry (20x) and Immunofluorescence (10x) of Aβ in cortex and hippocampus; **(B)** Congo red staining (labeled amyloidosis) in cortex and hippocampus(40x); **(C)** Expression of APP/Aβ1-42 and the short APP fragments Aβ1-42 were determined by Western blot. **(D-E)** Quantitation of Western blots. **p<0.01 3xTg vs WT; ^#^p<0.05 or ^##^<p: 0.01. 3xTg vs 3xTg+ART.

### Artemisinin reduced the deposition of Aβ and Tau in 3xTg mice

The 3xTg mice exhibited strong Aβ deposition, a neuropathological result of AD, in comparison to the WT mice (12 months old). Upon evaluating the effect of ART treatment on Aβ deposition, a significant reduction in extracellular Aβ deposition was seen in all of the examined cerebral regions of the ART+3xTg mice brain in comparison to 3xTg mice, which further validated the results (Fig. 2A). We got the similar result by Congo-red staining (Fig. 2B). Congo red is used for staining in amyloidosis, the results of Congo red staining showed that there were more Aβ deposits in cortex and hippocampus of 3xTg mice, while the Aβ deposits in cortex and hippocampus were significantly reduced after treatment with artemisinin. The result was further confirmed by western blot as presented in Figure 2C-E.

**Figure 3.**
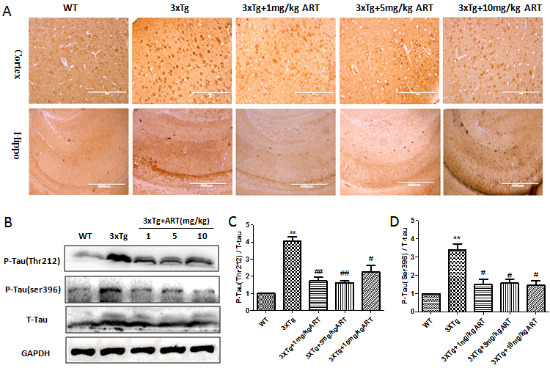
**Artemisinin reduced Tau deposition in aged 3xTg mice**. **(A)** Immunohistochemistry (Cortex 20x, hippo10x) of p-Tau in cortex and hippocampus. **(B)** Expression level of p-Tau was detected by Western blot. **(C-D)** Quantitation of Western blots in B. **p<0.01 3xTg vs WT; ^#^p<0.05 or ^##^<p: 0.01. 3xTg vs 3xTg+ART.

The hyperphosphorylation of Tau is another prominent feature that is responsible for neuronal death during AD [38]. In order to understand the underlying action mechanism of ART on cognitive improvement, we checked the tau phosphorylation in the 3xTg mice brain by IHC and western blots. As shown in Figure 3A, there was significant reduction in phosphorylated tau levels in the ART treated mice was recorded when compared to 3xTg mice. The result was confirmed by western blot (Fig. 3B-D). Overall, ART treatment resulted in a reduction in tau phosphorylation.

### Artemisinin attenuated the activation of glial cells and expression of inflammatory molecules in 3xTg mice

**Figure 4.**
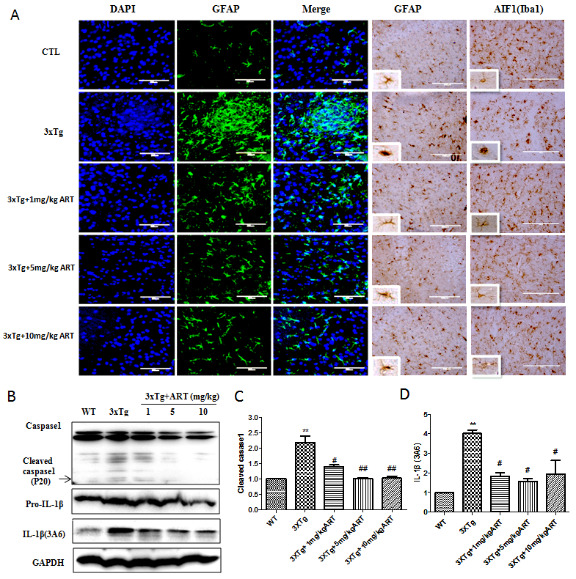
**Artemisinin attenuated the activation of glial cells, caspase activation and the expression of inflammatory molecules IL-1β in the brain of aged 3xTg mice**. **(A)** Immunofluorescence and Immunohistochemistry of GFAP (Astrocyte marker) and AIF1 (Iba1, microglia marker) (40x). **(B)** Western blot analysis of the effect of ART on IL-1β and caspase1. **(C-D)** Quantitation of cleaved caspase1 and IL-1β in B. **p<0.01 3xTg vs WT; ^#^p<0.05 or ^##^<p: 0.01. 3xTg vs 3xTg+ART.

Inflammation is another important pathophysiological feature of AD, accompanied by activation of glial cells and release of inflammatory factors [39, 40]. Overactivated astrocytes cells and microglia cells can stimulate neuroinflammatory reactions. Astrocyte activation is indicated by the emergence of a hypertrophic soma and processes and is often appear with an increase in the expression of GFAP, which is a major intermediate filament protein exclusive to astrocytes [8, 41]. Therefore, in current study we checked whether the expression of its specific marker GFAP changed in various treatment groups. The data displayed a significant increase in GFAP immunoreactivity in the 3xTg mice in comparison to the WT mice (Fig. 4A). However, ART treatment significantly decreased the GFAP immunoreactivity in cortex area, suggesting that ART may have anti-inflammatory effect.

In order to further confirm whether ART reduces inflammation, marker of microglia (Iba1) was also checked (Fig. 4A). Under normal conditions, the morphology of microglia cells is in a branched form, which can secrete growth factors to maintain the survival of neurons. After excessive activation, the branches of microglia shorten or even disappear, and secrete inflammatory factors leading to nerve cell death. From the result we found that ART treatment significantly improved microglia morphology in 3xTg mice. In support of these results, ART brought down the levels of the brain inflammatory markers IL-1β and cleaved caspase 1 which were found increased in 3xTg mice by western blot (Fig. 4B-D). The above results suggested that the therapeutic effect of ART in the 3xTg mice is due to both neuroprotective and anti-inflammatory effects.

### ART attenuated the histopathological changes and decreased neuronal apoptosis in 3xTg mice by activation of ERK

**Figure 5.**
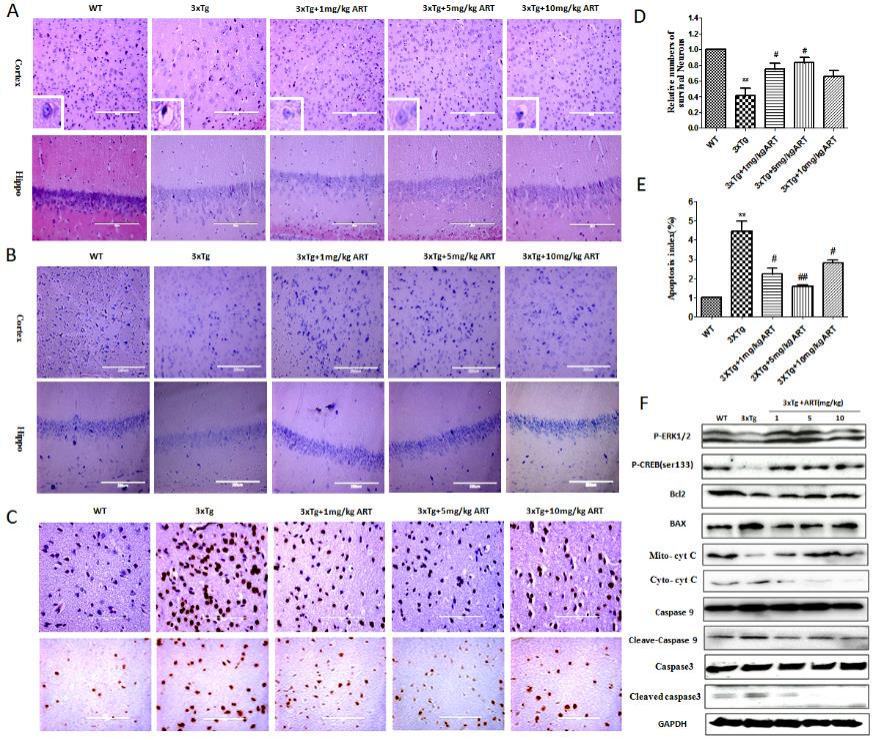
**Artemisinin attenuated the histopathological changes and decreased neuronal apoptosis in 3xTg mice by activation of ERK**. **(A)** HE staining showed the histopathological changes of neurons in 3xTg mice (40x). The cells were vacuolated, and ART increased cell number and reduced cell vacuolation; (B) Neuronal cell function was then examined using Nissl staining and our result indicated that artemisinin significantly improved neuronal function of 3xTg mice (40x); (C) Apoptosis determined by TUNEL staining in cortex of wild type and 3xTg mice treated as indicated (40x) . **(D)** Statistical analysis results of Nissl staining. **(E)** Quantitation of the deoxynucleotidyl transferased UTP nick-end labeling (TUNEL) staining in C. **(F)** The expression of p-ERK1/2, p-CREB, Bax, Bcl-2, Cytochrome C, cleaved caspase 9 and cleaved caspase-3 (active form of caspase 3) and GAPDH were detected by Western blot. **p<0.01 3xTg vs WT; ^#^p<0.05 or ^##^<p: 0.01. 3xTg vs 3xTg+ART.

Histopathological changes were recorded in HE-stained images of hippocampus and cortex of mice from WT, 3xTg and 3xTg+ART groups. The results showed that injured neurons in 3xTg mice were darkly stained and also exhibited vacuolar bodies. Cells were arranged in disorder with slight change and neuron loss was also seen. In contrast, treatment with ART significantly restrained the histopathological damage, improved the vacuolar bodies (Fig. 5A). When neurons are damaged, Nissl body can get reduced or even disappeared, and the cells get deeply stained with eosin. Nissl staining showed that in the WT group the Nissl bodies were large and numerous, indicating that the protein synthesis activity was stronger in nerve cells, whereas the number of Nissl bodies in 3xTg mice was reduced, the staining was shallow, and the staining was unclear. This condition was improved after ART treatment (Fig. 5B and D). We further observed the effect of ART on brain cortical neuronal apoptosis by TUNEL staining. The number of apoptotic brain cortical neurons was significantly decreased following treatment of 3xTg mice with ART, relative to untreated 3xTg mice (Fig. 5C and E).

Studies indicated the involvement of ERK in the inhibition of apoptosis. Here, the expression of p-ERK1/2 and p-CREB was determined to evaluate the treatment effect of ART in 3xTg mice. The expression level of p-ERK1/2 and P-CREB was significantly increased in 3xTg+ART group compared with 3xTg group (Fig. 5F). We tested the expression of apoptosis regulator in different treatment groups also. The expression levels of Bax, Bcl-2, Cytochrome C, caspase-9 and caspase-3 were detected by western blotting (Fig. 5F). The expression of proapoptotic factors Bax, Cytochrome C, cleaved-caspase-9 and cleaved caspase-3 was upregulated while that of Bcl-2 (an inhibitor of apoptotic proteins) was downregulated in 3xTg mice in comparison to the WT group. Treatment with ART increased the ratio of Bcl-2 /Bax compared with the 3xTg group. These results indicated that ART reduced brain neuronal apoptosis may through ERK/p-CREB/Bcl2 axis.

### ART attenuated the cell viability induced by Aβ_1-42_ in SY5Y cells

**Figure 6.**
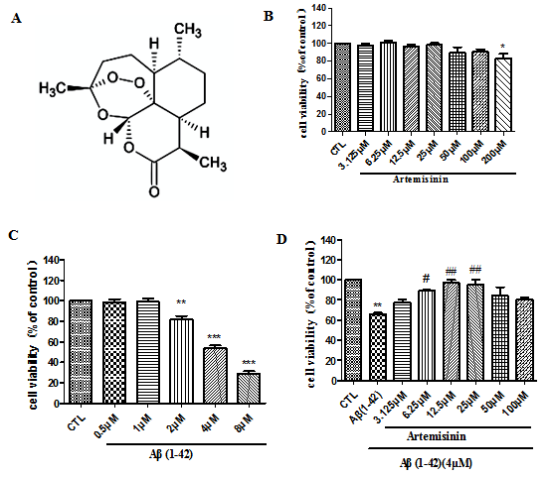
**Artemisinin concentration- and time-dependently reversed the decrease in cell viability**. **(A)** Chemical structure of ART. **(B)** The cytotoxicity of ART, cells were treated with ART (3.125-200μM) for 24h and cell viability was measured using the MTT assay. **(C)** The cytotoxicity of Aβ_1-42_. Cells were treated with Aβ_1-42_ (0.5-8μM) for 24h and cell viability was measured using the MTT assay. **(D)** The effect of ART on cell viability. Cells were incubated with ART at indicated concentrations with or without 4μM Aβ_1-42_ for another 24h and cell viability was measured using the MTT assay. *P<0.05 or **P<0.01, or ***P<0.001, CTL vs Aβ_1-42_; ^#^P<0.05 or ^##^P<0.01 Aβ_1-42_ vs Aβ_1-42+_ART.

SH-SY5Y cells were incubated with different concentrations of ART or Aβ_1-42_ for 24h and MTT assay was used to determine the cell viability. ART treatment did not induce any cytotoxicity in SH-SY5Y cells up to a concentration of 200μM (Fig. 6B). Aβ_1-42_ caused significant cytotoxicity in SH-SY5Y cells starting at 2μM in comparison to the control group (Fig. 6C). The cell viability was almost half, so we chose 4μM Aβ_1-42_ for all of the following experiments. In order to examine the protective effects of ART, SH-SY5Y cells were pretreated with different dose of ART for 2h before exposing to Aβ_1-42_ for another 24h. Results showed that pre-treatment with 12.5μM ART can significant reduce Aβ_1-42_-induced cell death (Fig. 6D).

### Artemisinin pretreatment attenuated Aβ_1-42_ -induced apoptosis by reducing ROS level and restored the mitochondrial membrane potential in SH-SY5Y cells

**Figure 7.**
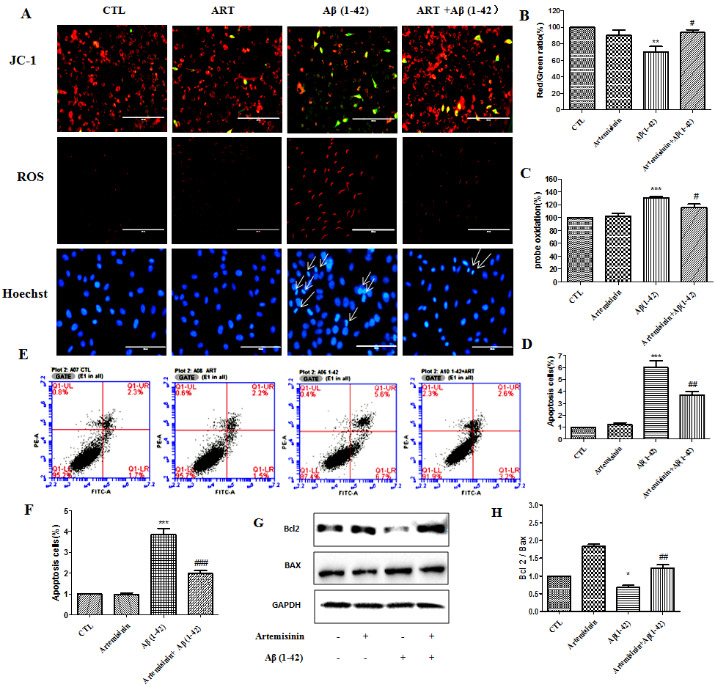
**Artemisinin restored the mitochondrial membrane potential, and decreased ROS accumulation and reduced apoptosis induced by Aβ_1-42_ in SH-SY5Y cells**. **(A)** Cells were pretreated with 12.5μM ART for 120min and then induced with or without 4μM Aβ_1-42_ for a further 24h. The decline in the mitochondrial membrane potential was reflected by the shift of fluorescence from red to green indicated by JC-1. Intracellular ROS level was measured by the CellROXs Deep Red Reagent. Nuclear morphology measured by Hoechst staining (40x). **(B-D)** Statistical analysis results of JC-1, ROS and Hoechst staining in A. **(E)** Photographs of representative cultures measured by Flow cytometry. **(F)** Statistical analysis results of Flow cytometry in E. **(G)** Expression of Bax, Bcl-2, and GAPDH were detected with Western blotting. **(H)** Quantification of representative protein band from Western blotting in G. *P<0.05 or **P<0.01, or ***P<0.001, CTL vs Aβ_1-42_; ^#^P<0.05 or ^##^P<0.01 or ^###^P<0.001 Aβ_1-42_ vs Aβ_1-42+_ART.

SH-SY5Y cells pretreated with or without 12.5 μM ART for 2h were further treated with Aβ_1-42_ for 24h. The mitochondrial membrane potential (△ψm) in SH-SY5Y cells was assessed by calculating the red/green fluorescent intensity ratio upon JC-1 staining. Cellular oxidative stress was assessed by CellROXs Deep Red Reagent. The results revealed that ART pretreatment significantly hindered the decline of △ψm and brought down the intracellular ROS production induced by Aβ_1-42_. Nuclei condensation was seen in SH-SY5Y cells after exposure to 4μM Aβ_1-42_ by Hoechst 33342 staining assay. However, pre-treatment with 12.5 μM ART significantly improved these changes (Fig. 7A - D). Cell apoptosis was further verified using flow cytometry for Annexin V-FITC/PI-positive cells and the results from these experiments indicated that Aβ_1-42_ exposure markedly increased apoptosis in SH-SY5Y cells, while 12.5μM ART pretreatment significantly reduced the apoptosis caused by Aβ_1-42_ (Fig. 7E and F). Western blotting revealed that ART pretreatment significantly reduced the expression level of Bax and increased the level of Bcl2 (Fig. 7G and H).

**Figure 8.**
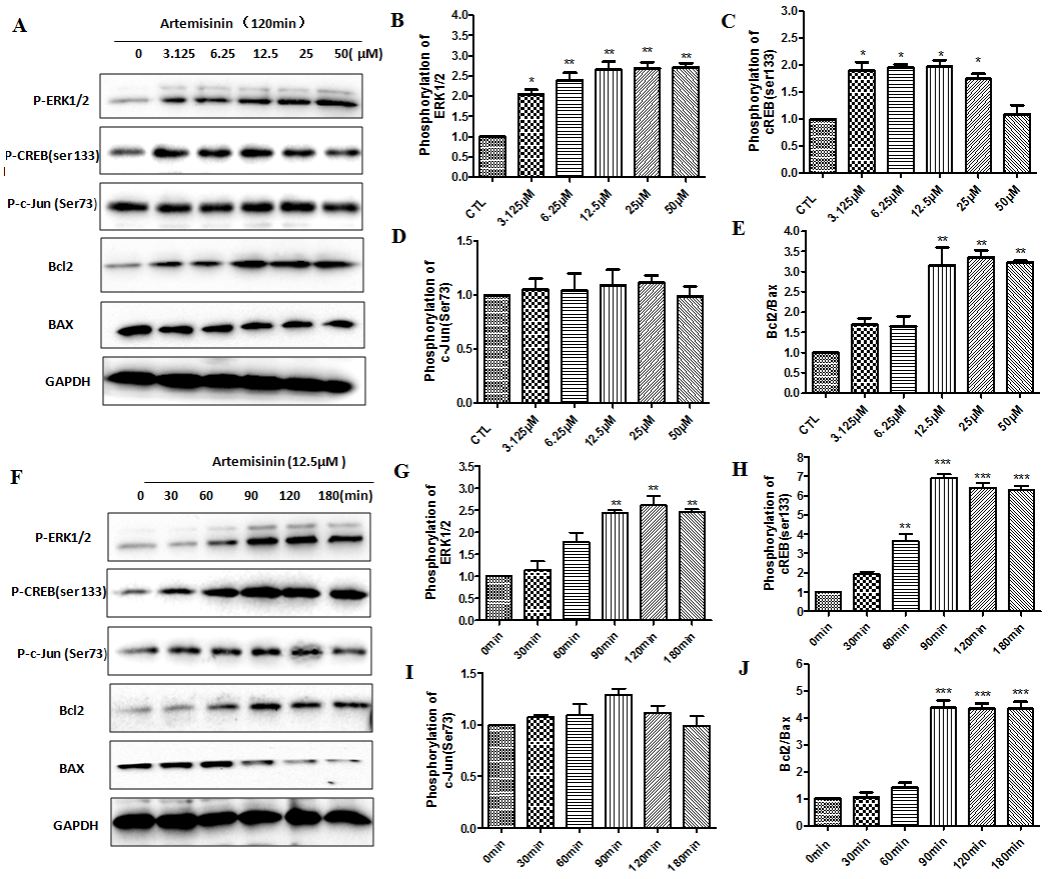
**Artemisinin increased phosphorylation of ERK and CREB (ser133) in SH-SY5Y cells. (A) The SH-SY5Y cells were treated with ART for 120 min at different concentrations (3.15, 6.25, 12.5, 25 and 50 μM) and the expression of P-ERK1/2, p-CREB, P-C-Jun, Bcl2, Bax and GAPDH were detected by Western blot**. **(B-E)** Statistical analysis results of A. **(F)** The SH-SY5Y cells were treated with ART for different times (0, 30, 60, 90, 120 and 180 min) at 12.5 μM and the expression of P-ERK1/2, p-CREB, P-C-Jun, Bcl2, Bax and GAPDH were detected by Western blot. **(G-J)** Statistical analysis results of F. *P<0.05 or **P<0.01, or ***P<0.001.

### ERK involved the protective effects of artemisinin in SH-SY5Y cells

Figure 5F have shown that ART stimulated the activation of ERK/CREB pathway in 3xTg mice brain, so we used western blot to find out whether the ERK/CREB signaling pathway is regulated by ART in SH-SY5Y cells also. Results showed that within 180 min, ART gradually raised the phosphorylation intensity of ERK1/2 and CREB in SH-SY5Y cells in a time- and dose- dependent manner. But we have not found that ART has a significant change in phosphorylation intensity of c-Jun. These results indicated that ART might be activating ERK/CREB survival signaling. Similarly, a time- and dose- dependent increase of Bcl-2/Bax ratio upon ART treatment was observed by Western blot (Fig. 8A-J). Taken together, our results demonstrated that artemisinin reduced apoptosis through activation of ERK/CREB/Bcl-2 axis in SH-SY5Y cells.

**Figure 9.**
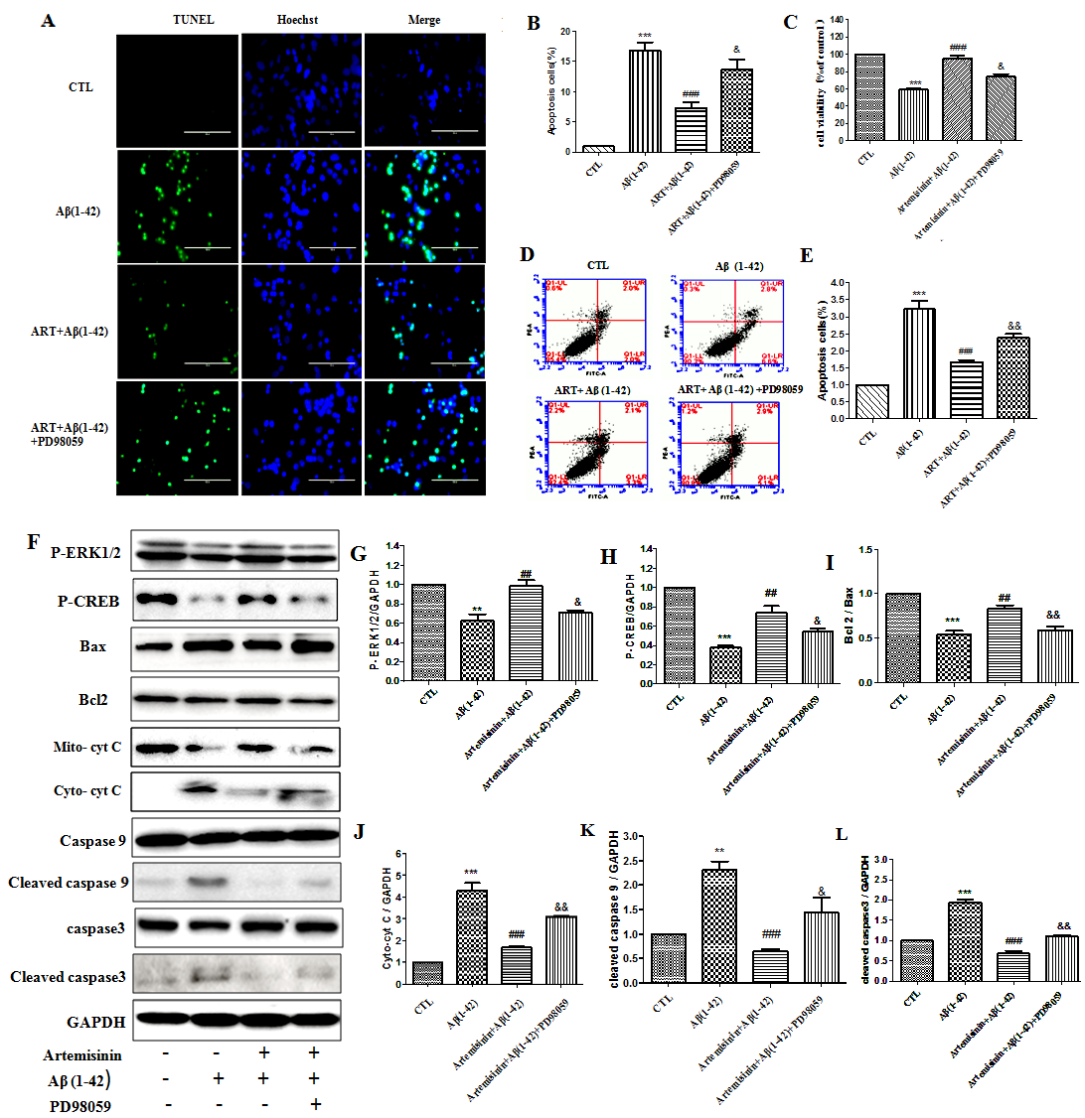
**ERK/CREB pathway mediated the protective effects of artemisinin in SH-SY5Y cells**. **(A-B)** Cells, pretreated with 25 μM PD98059 (ERK inhibitor) for 60 min, were incubated with 4 μM Aβ_1-42_ in the presence or absence of 12.5μM ART. Apoptosis was measured by Tunel staining (40x). **(C)** Cell viability was measured by MTT assay. **(D-E)** Apoptosis was measured by Flow cytometry. **(F)** P-ERK1/2, P-CREB, Bax, Bcl2, Cytochrome C, cleaved caspase9 and cleaved caspase3 were measured by Western blot. **(G-L)** Statistical analysis results of P-ERK1/2, P-CREB, Bax, Bcl2, Cytochrome C, cleaved caspase9, cleaved caspase3. *: Difference between the Aβ_1-42_ group and WT groups; #: Difference between the Aβ_1-42_ and other groups; &: Difference between the ART+Aβ_1-42_ and other groups; **P<0.01, ***P<0.001, ^##^P<0.01, ^###^P<0.001, ^&^P<0.05, ^&&^P<0.01.

To further confirm whether ERK is associated with the survival promoting effect of ART on cell apoptosis induced by Aβ_1-42_, we pretreated the cells with PD98059 (a specific inhibitor of ERK) for 60min and then the cells were treated with ART for 2 h followed by Aβ_1-42_ for another 24h. TUNEL staining, Flow cytometry and MTT results showed that pretreatment of PD98059 blocked the protective effects of ART (Fig. 9A-E). Otherwise, the recovery of the mitochondrial membrane potential and decreased the intracellular ROS were reversed after PD98059 added (Supplementary Fig. 1). Similar results were obtained from Western blot which showed that ART was not able to suppress the expression of apoptosis regulators: Cytochrome C, cleaved caspase-9, cleaved caspase 3 and increased the expression of p-CREB, Bcl-2/Bax ratio in the presence of the ERK pathway inhibitor PD98059 (Fig. 9F-J), which suggested that ERK/CREB signaling is involved in the protective effects of ART against Aβ_1-42_ induced toxicity.

## DISCUSSION

AD is one of the neurodegenerative disorders that affects older population and is clinically characterized by progressive loss of memory along with decline in multiple cognitive abilities [42, 43]. Aβ accumulation plays a crucial role in AD which could cause a number of important pathological changes such as: oxidative stress, inflammation, defects and alterations in cholinergic neurons, synaptic deficit, proliferation of reactive astrocytes, and neuronal death [44, 45]. As the multifactorial nature of AD, the search for Chinese herbal compounds with a wide spectrum of neuroprotective activities, hold very encouraging future for the treatment of AD [46]. ART, widely used in the clinic as an anti-malarial drug, has exhibited neuroprotective effects in various neuronal cells [47, 48]. However, the mechanism is still not clear and protective effects of ART on cognitive impairment and pathology *in-vivo* are not well known so far. 3xTg mice develop Aβ and tau pathology, as well as neuroinflammation and cognitive deficits. In order to check all the pathological features of AD, we chose one-year-old mice to perform our experiments. In this study, we demonstrated preclinical *in-vitro* (SH-SY5Y) and *in-vivo* (3xTg) neuroprotective correlations (IVIVC) for ART.

It is clear from Morris water maze results, that, ART can lessen the escape latency and path length of 3xTg mice, suggesting that ART could recover the learning and spatial memory capacity during AD. The hallmarks of AD are extracellular accumulation of Aβ and intracellular NFT [49, 50]. Deposition of Aβ and Tau in brain is the primary characteristic as well as drug target for the diagnostic and therapeutic intervention of AD[51]. Aβ accumulates and deposits into soluble fibrils, oligomers, and senile plaques, which is the main pathological reason for synaptic malfunction and neuronal network disruptions, ultimately causing massive atrophy of the brain and cognitive deterioration in AD patients and 3xTg mice[52, 53]. Our result showed that ART treatment significantly reduced the rising numbers of senile plaques in the cerebral cortex and hippocampus of 3xTg mice.

Results from the study on transgenic mice model for Amyloid precursor protein have shown that Aβ can continue to activate inflammatory response, including excessive activation of glial cells and activation of cytokines[54]. GFAP is a primary intermediate filament protein which is specific to astrocytes. The increased GFAP is usually considered as an indicator of gliosis or a comparatively slow-developing index of neural damage linked with old age and the onset of AD pathology[55, 56]. We found that the GFAP activity was significantly decreased with the treatment of ART in 3xTg mice. Microglia (MG) is the main immune cell of the CNS and widely distributed in the brain. Overactivated microglia will release a large number of pro-inflammatory and other neurotoxic substances which will cause varying degrees of damage to neurons. Our result showed that ART treatment significantly improved microglia morphology in 3xTg mice. In addition, ART reduced the release of main inflammatory factors, further supporting its anti-inflammatory effects.

Previous reports have suggested that mitochondrial dysfunction, oxidative stress, and apoptosis may play important role in the overall process of AD. Aβ can result in the decrease in the △ψm of atrocities via activation of NADPH oxidase, causing oxidative stress and leading to neuronal death[57, 58] . Aβ also damages mitochondria causing dysfunction of mitochondrial complexes I and IV, which result in reactive oxygen species (ROS) overproduction and adenosine triphosphate (ATP) depletion. ROS increase in turn leads to mitochondrial permeability transition pore (MPTP) open which increases mitochondrial damage [59, 60]. Damaged mitochondria release Cytochrome C, further activating the caspase family and triggering apoptosis. The Caspase family exerts a crucial role in mediating apoptosis, where Caspase-3 is the main executive molecule functioning in multiple apoptotic signaling pathways. Caspase-3 usually exists in the cytoplasm in the form of zymogens (32KD), which is activated upon apoptosis, and cleaves the corresponding cytoplasm nucleus substrate, eventually leading to apoptosis. Bcl2/Bax family is a major mitochondrial protein. The ratio of Bcl2/Bax is a "molecular switch" that initiates apoptosis [61-63]. In present study, the apoptosis regulator Cytochrome C, caspase-9, caspase-3 activity was inevitably decreased and ratio of Bcl2/Bax was slightly increased with ART treatment in 3xTg mice and SH-SY5Y cells. Similar results were obtained by TUNEL staining in the brain of 3xTg mice. In addition, ART treatment of Aβ-induced cell death reduced the production of ROS, corrected mitochondrial membrane potential in SH-SY5Y cells.

ERK pathway plays central role in the initiation and regulation of many accelerated cellular processes such as proliferation, differentiation, and survival [64]. It has been shown that neuroprotection in AD could result from the decreasing level of oxidative stress mediated by ERK activation [65]. In present study, the rise in ERK1/2 phosphorylation level, after treatment with ART, was seen both in 3xTg mice and SH-SY5Y cells. After ART intervention, the expression of Bax was brought down, while the expression level of Bcl2 was enhanced significantly. Furthermore, our results demonstrated that ART failed to suppress Aβ_1-42_-induced cytotoxicity, the increase of Bcl2/Bax ratio and caspase3 activity in the presence of PD98059 which is an ERK inhibitor. According to these results, ART regulated the protective effects against Aβ_1-42_-induced damage by ERK/CREB pathway. Thus the inhibition of ERK could play important role in therapeutics and pathophysiological of AD.

**Figure 10.**
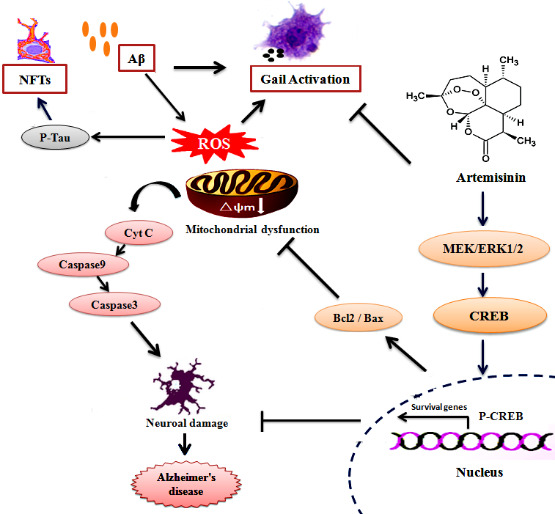
**The possible mechanism of artemisinin mediated neuroprotection against Aβ_1-42_-induced injury in neuronal cells and in 3xTg mice**. Artemisinin stimulated ERK1/2 phosphorylation in neuronal cells and the brain of 3xTg mice, which results in activation of CREB/Bcl-2 survival pathway and inhibit apoptosis pathway. This process may reduce oxidative stress, correction of mitochondrial dysfunction. In addition, artemisinin also reduced the inflammatory release and reduced the deposition of amyloid plaques and neurofibrillary tangles.

Our study suggests that ART can protect neuronal cells from Aβ_1-42_-induced cell damage *in-vivo* and *in-vitro* of AD models via, at least in part, the activation of the ERK/CREB pathway and inhibition of apoptosis pathway. Furthermore, ART can alleviate the learning and spatial memory destruction in 3xTg mice. At the same time, ART reduced the Aβ and Tau deposition and also reduced the inflammatory response in the brain of 3xTg mice (Fig. 10). Therefore, therapeutic intervention with ART could be useful for AD patients.

## Supplementary Materials

The Supplementary data can be found online at: www.aginganddisease.org/EN/10.14336/AD.2019.0813.


