# Supplementary material for: Artemisinin Improved Neuronal Functions in Alzheimer's Disease Animal Model 3xtg Mice and Neuronal Cells via Stimulating the ERK/CREB Signaling Pathway
Source: Aging Dis. 2020 Jul 23;11(4):801–19. doi: 10.14336/AD.2019.0813 (PMC7390534; doi:10.14336/AD.2019.0813)
Supplement: Supplementary file 1 [file AD-11-4-801-suppl.pdf]

SUPPLEMENTARY DATA

Artemisinin Improved Neuronal Functions in Alzheimer's Disease Animal Model 3xtg Mice and Neuronal Cells via Stimulating the ERK/CREB Signaling Pathway

Xia Zhao, Shuai Li, Uma Gaur, Wenhua Zheng*

Center of Reproduction, Development & Aging and Institute of Translation Medicine, Faculty of Health Sciences, University of Macau, Taipa, Macau, China

© 2019. Zhao X et al. Published online at http://www.aginganddisease.org/EN/10.14336/AD.2019.0813

SUPPLEMENTARY DATA

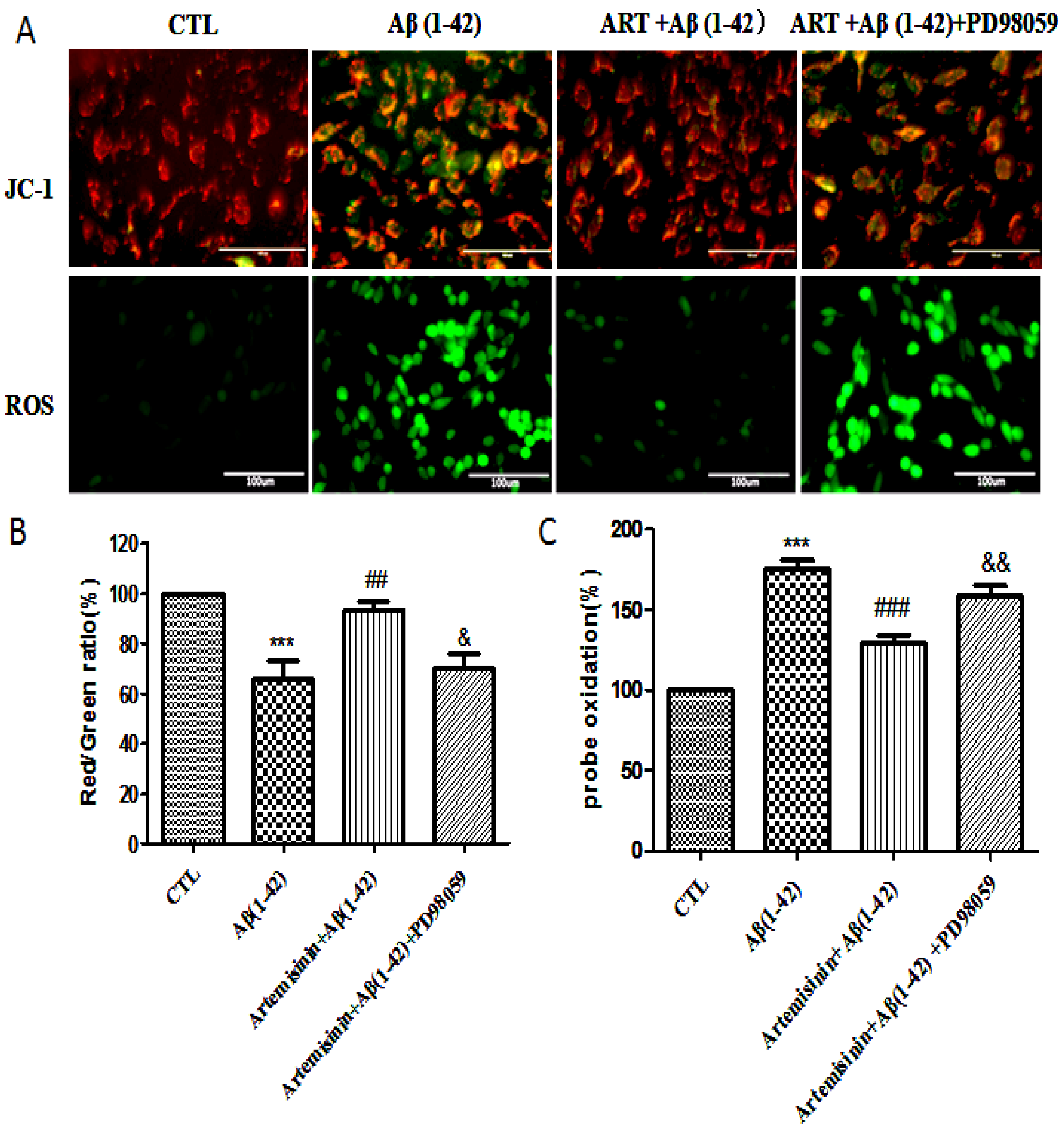

Supplementary Figure 1. ERK inhibitor PD98059 reverses the protective effect of Artemisinin on ROS and mitochondrial membrane potential (△ψm) in SH-SY5Y cells. (A) Cells, pretreated with 25 μM PD98059 (ERK inhibitor) for 60 min, were incubated with 4 μM $A\beta_{1\text{-}42}$ in the presence or absence of 12.5μM ART. The mitochondrial membrane potential was reflected by the shift of fluorescence from red to green indicated by JC-1. The fluorescent images represent the intracellular ROS level as determined by the DCFH-DA Reagent. (B) Red to green fluorescence intensity ratio (increase of mitochondrial membrane potential). (C) Quantitation of the percentage of intracellular ROS level. *: Difference between the $A\beta_{1\text{-}42}$ group and WT groups; #: Difference between the $A\beta_{1\text{-}42}$ and other groups; &: Difference between the ART+$A\beta_{1\text{-}42}$ and other groups; ***P<0.001, $^{\#\#}$P<0.01, $^{\#\#\#}$P<0.001, $^{\&}$P<0.05, $^{\&\&}$P<0.01.

© 2019. Zhao X et al. Published online at http://www.aginganddisease.org/EN/10.14336/AD.2019.0813
